# Readmission predictors at three years after a manic episode.

**DOI:** 10.1192/j.eurpsy.2024.114

**Published:** 2024-08-27

**Authors:** A. Giménez-Palomo

**Affiliations:** ^1^Bipolar and Depressive Disorders Unit, IDIBAPS; ^2^Institute of Neuroscience, Hospital Clínic de Barcelona, Barcelona, Spain

## Abstract

**Abstract:**

In this section, the speaker will present the results from a recent longitudinal study performed by the work group, in which a cohort of 265 patients admitted with a manic episode were followed up during three years after hospital discharge to identify acute readmissions due to affective relapses. The study of different sociodemographic and clinical variables potentially implicated in a higher risk of readmission over three years is presented, including adherence to treatment, substance use, number of previous episodes, family history, predominant polarity, treatments used and number of visits to the Emergency Department.

**Disclosure of Interest:**

None Declared

